# The Effects of Kefir on the Human Oral and Gut Microbiome

**DOI:** 10.3390/nu17243861

**Published:** 2025-12-11

**Authors:** Eleni Grace Black, Andrea Bugarcic, Romy Lauche, Emad El-Omar, Fatima El-Assaad

**Affiliations:** 1 Microbiome Research Centre, School of Clinical Medicine, UNSW Medicine & Health, St George & Sutherland Clinical Campuses, Sydney, NSW 2217, Australiae.el-omar@unsw.edu.au (E.E.-O.); 2 National Centre for Naturopathic Medicine, Southern Cross University, Lismore, NSW 2480, Australia; andrea.bugarcic@scu.edu.au (A.B.); romy.lauche@scu.edu.au (R.L.)

**Keywords:** microbiome, kefir, gut microbiome, oral microbiome, probiotics

## Abstract

Kefir, a fermented probiotic drink made from milk, water, or plant-based ingredients, has gained significant attention as a dietary supplement. Originating from the Caucasus Mountains over three thousand years ago, kefir is believed to harbor a range of health benefits through its ability to alter the composition of microbial niches within the human body. These microbial niches are called microbiomes and encompass the collective community of microbial organisms, their genomes and environment. The modern commercialization of kefir has driven the need for high-quality research into its impact on the human microbiome and associated health outcomes; however, there is currently very limited scientific evidence supporting effects of kefir consumption on the human oral and gut microbiome. High-quality human clinical trials are essential to establish the safety and effectiveness of kefir before it can be advised for use in treating conditions linked to the oral and gut microbiota or metabolic health. This literature review aims to critically analyze recent studies investigating the effect of kefir consumption on the oral and gut microbiome, as well as its potential implications for human health. By examining kefir’s effects on these interconnected microbial ecosystems, we can better understand its potential and limitations as a functional food for promoting systemic health.

## 1. Introduction

The term ‘microbiome’ was initially coined in 1988 by Whipps et al. to describe the symphony of microbes existing in soils [1]. Since then, the term has expanded to include assemblies of microorganisms critical in the maintenance of human health and describes both the living microorganisms within an ecological niche as well as their respective genomes. The human gut microbiome is largely considered the most critical microbiome given its influence on metabolic health, chronic diseases and immune health, as well as its intricate communication with distal organs and organ systems [2,3]. The gut microbiome is typically rich and vast, carrying approximately 100 trillion microbes that span across hundreds to thousands of species [4,5]. This is in stark contrast to the oral microbiome, another niche within the human body with great influence on human health. While over 700 bacterial taxa have been reported in the oral cavity, the precise number of microbes is only 50–100 billion, dwarfed by that of the gut [6]. Nevertheless, the oral microbiome remains a critical factor in influencing human health due to its role in digestive and metabolic functions as well as its wider link to systemic health [6]. Probiotics have been consumed across the globe for millennia and are believed to promote human health by altering the balance of microbiota within a specific niche. Most probiotic products act mainly on the gut, containing live microorganisms that compete with pathogenic bacteria for nutrients and adhesion sites by producing antimicrobial substances such as bacteriocins and organic acids [7,8,9]. Additionally, by interacting with the host’s immune system, these microbes can reduce local and systemic inflammation and support gut barrier integrity [10,11,12]. While probiotics are naturally present in foods like yogurt, sauerkraut (fermented cabbage), and kimchi (fermented mixed vegetables), they are also available as dietary supplements in capsule or powder form. The ability of probiotics to influence microbial niches in the human body makes them a valuable tool for promoting overall health and preventing disease [13,14].

**Figure 1:**
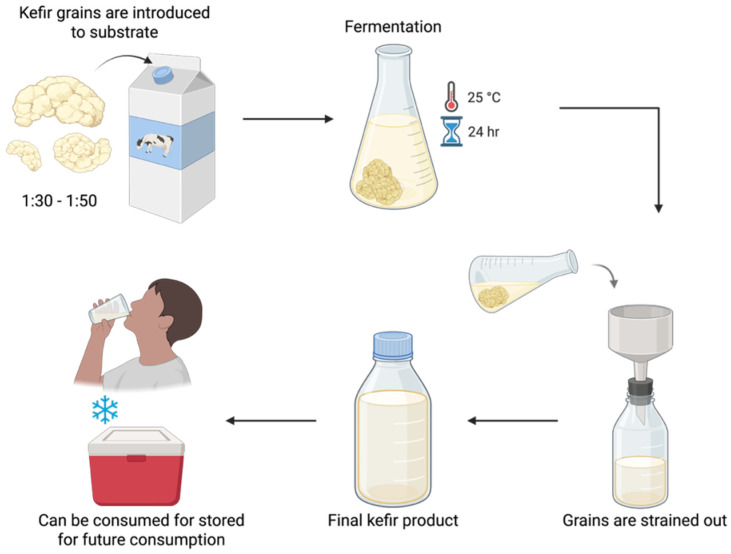
A simplified flowchart demonstrating commercial kefir production. Kefir grains are added to a base, either milk, water or plant-based beverage, in a ratio of 1:30 to 1:50 and the mixture undergoes fermentation at room temperature. After 24 h, the grains are strained out, and the remaining beverage can be consumed immediately or stored for later consumption. Created in BioRender. El-Assaad, F. (2025).

Kefir is a probiotic milk beverage that originated in the Caucasus mountains over 3000 years ago [15]. It is produced using kefir grains, which are complex symbiotic communities of lactic acid bacteria, acetic acid bacteria and yeast embedded in a polysaccharide matrix. The grains are introduced to milk, typically cow, and initiate a fermentation process by which the milk becomes thickened and slightly sour. In commercial kefir production practices (Figure 1), milk is inoculated with kefir grains in a ratio of 1:30 to 1:50, and the mixture is then left to ferment for up to 24 h at room temperature [16,17]. Finally, the kefir grains are strained out, and the remaining liquid can be consumed immediately or stored at low temperatures for future consumption [17,18]. Kefir can also be made using goat and sheep milk, or from vegan alternatives such as soymilk and water [19,20]. As the commercial kefir market has expanded, so too have the options for low-lactose and vegan alternatives, thereby allowing individuals with specific dietary requirements to consume the beverage. However, the exact microbial composition of kefir beverages, and therefore the health benefits they confer, may largely depend on the liquid substrate used during fermentation [19].

Indeed, an important consideration in kefir research is the inherent heterogeneity of kefir itself. Kefir composition varies considerably depending on multiple factors, including the microbial profile of the starter grains, fermentation time, and fermentation temperature [21,22,23,24]. These production variables can significantly influence the final microbial species present in the beverage, their relative abundances, and the concentration of bioactive metabolites produced during fermentation. Consequently, the specific health effects observed in one study may not be directly comparable to those in another, as different kefir preparations may exert distinct effects on the oral microbiome. For this reason, evidence on changes in the gut and oral microbiome following kefir consumption can largely differ [25,26].

**Figure 2:**
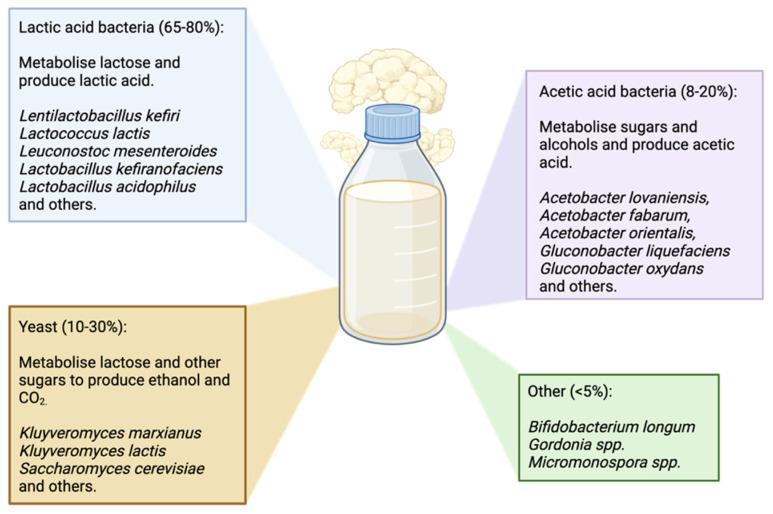
Microbial composition of kefir: lactic acid bacteria (65–80%), yeast (10–30%), acetic acid bacteria (8–20%) and others (<5%). Created in BioRender. El-Assaad, F. (2025).

The microbial composition of kefir is largely dominated by lactic acid bacteria (LAB), representing 60–83% of the microbial population [27,28,29,30] (Figure 2). LAB play a crucial role in the fermentation of milk substrates by metabolizing lactose and producing lactic acid. During this process, LAB also produce carbon dioxide, acetaldehyde, bacteriocins, cathelicidin and hydrogen peroxide, which have been demonstrated to attenuate and/or eradicate common enteric pathogens [31,32]. *Lentilactobacillus kefiri*, *Lactococcus lactis* and *Leuconostoc mesenteroides* are among the most abundant LAB present in kefir [33,34]. *L. kefiri* and *L. mesenteroides* survive passage to the gut and adhere to the epithelial lining, a critical feature of potential probiotics [35,36]. In the gut, these species display antibacterial and antifungal properties [35,36,37]. *L. kefiri* has also been found to bind toxic metals and mycotoxins, boasting its potential for future use in emergency toxicology [38,39]. *L. mesenteroides* can produce linoleic acid, a compound with antiatherogenic, anti-inflammatory, and anticarcinogenic properties [36]. *L. lactis*, in comparison, can produce conjugated linoleic acid but is unable to adhere to intestinal epithelium to colonize the gut [40,41]. Despite its transient presence in the gut, *L. lactis* can confer benefits to the host by producing biological compounds with immunomodulatory, antibacterial and antihypertensive effects [41]. It is this characteristic of *L. lactis* that earned it the term ‘cell factory’, with several studies showing its potential as a vehicle to deliver therapeutics and vaccines [42,43,44,45,46]. Other LAB species present in kefir include *Lactobacillus acidophilus*, *Lactobacillus delbrueckii*, *Lactobacillus helveticus*, *Lactobacillus johnsonii*, *Lentilactobacillus parakefiri*, *Lentilactobacillus sunkii*, *Lacticaseibacillus rhamnosus*, *Lacticaseibacillus casei*, *Lacticaseibacillus paracasei*, *Lactiplantibacillus plantarum*, *Levilactobacillus brevis*, *Limosilactobacillus fermentum*, and *Limosilactobacillus reuteri* [47,48]. However, the proportion and even the presence of each bacterial species in kefir varies across regions, substrates used and manufacturers [47,49,50].

The remaining 17–40% of microbial load in kefir is largely distributed amongst acetic acid bacteria (AAB) and yeast [16,29,30,47]. AAB such as *Acetobacter lovaniensis*, *Acetobacter fabarum*, *Acetobacter orientalis*, *Gluconobacter liquefaciens* and *Gluconobacter oxydans* have been identified in kefir [51]. These bacteria play a role in the fermentation of kefir along with LAB, producing acetic acid, a key short-chain fatty acid (SCFA) in the gut [52,53,54]. Acetic acid and its metabolites have local effects, such as an increase in ileal motility and colonic blood flow, as well as maintenance of epithelial homeostasis [55,56,57]. Recent studies also highlight its influence on systemic health, through modulation of host inflammation, energy expenditure and appetite [57]. Yeasts present in kefir include both lactose-fermenting species, such as *Kluyveromyces marxianus* and *Kluyveromyces lactis*, and non-lactose fermenting species, such as *Saccharomyces cerevisiae* [30,58,59]. *S. cerevisiae*, *K. marxianus* and *K. lactis* produce ethanol and carbon dioxide, contributing to kefir’s distinct flavor and mild effervescence [60,61]. Yeasts present in kefir are generally safe and well-tolerated for human consumption; indeed, many yeasts are considered probiotics as they support immune function and gut health [62]. Specifically, *S. cerevisiae var. boulardii* exhibits antimicrobial, anticarcinogenic, antioxidant and anti-inflammatory properties [63] and has been documented to cure or prevent Crohn’s disease [64] and irritable bowel syndrome [65]. Evidence suggests that *S. cerevisiae* may also enhance the probiotic potential of LAB via coaggregation and adhesion to epithelial cells [66], which is a particularly notable finding when considering the overall microbial profile of kefir. Kefir also contains *Bifidobacterium longum*, belonging to the *Bifidobacteriaceae* family. While neither LAB nor AAB, this species is known to have probiotic characteristics [67,68].

The unique combination of these microbial components is believed to contribute to kefir’s diverse range of potential health benefits, including improved gut health and management of various diseases [28,33,48]. The increasing popularity of kefir worldwide warrants further research into the impact of its consumption on human health. This review explores the current evidence the effect of kefir consumption on the oral and gut microbiome, the implications for human health and identifies key areas for future research.

## 2. Methods

A search was conducted using PubMed and Cochrane Library covering the period from January 2010 to February 2025. Search terms included “kefir” AND (“gut microbiota” OR “gut microbiome” OR “oral microbiota” OR “oral microbiome” OR “salivary”). A total of 126 results were retrieved, and 9 studies were included [69,70,71,72,73,74,75,76,77].

Studies were included if they met the following criteria: (1) randomized controlled trials, interventional studies, or safety/feasibility studies; (2) initial publication between January 2010 and December 2024 (3) methods included identification of gut or oral microbiota via DNA extraction and sequencing or culturing methods; and (4) outcomes included changes in the diversity or relative abundance or count of gut or oral microbiota. Studies were excluded if they were non-human studies, reviews, meta-analyses, case reports, or studies not available in English. Studies on any type of kefir (cow, goat, soy, water, etc.) were eligible for inclusion. Most studies were excluded as they were not randomized controlled trials, interventional studies, or safety feasibility studies, and did not measure changes in the diversity or relative abundance or count of gut or oral microbiota.

One further study was identified in the references of another included study [78]. Four studies were included examining the impact of kefir consumption on the oral microbiome, and six were included for the gut microbiome [69,70,71,72,73,74,75,76,77,78].

## 3. Results

Six relevant studies examining the gut microbiome and four examining the oral microbiome were identified (Table 1 and Table 2).

## 4. Discussion

### 4.1. Changes in the Gut Microbiome Induced by Kefir Consumption

Kefir consumption induces variable changes in the gut microbiome across different populations. In healthy adults, Walsh et al. [72] found that a subset of individuals experienced an increase in relative abundance of *Lactococcus raffinolactis*, a LAB present in milk kefir products. This change was not statistically significant, and there was no evidence of changes in microbial diversity between the three groups included in the study. In comparison, Bellikci-Koyu et al. [73] observed a significant increase in the relative abundance of *Actinobacteria* in individuals with metabolic syndrome. Yılmaz et al. [74] reported an increased relative abundance of *Lactobacillus* in individuals with IBD. Among critically ill patients admitted to hospital, Gupta et al. [71] noted an increase in the Gut Microbiome Wellness Index (GMWI) despite decreases in diversity and richness of gut microbiota due to antibiotic use. In contrast, Dazıroğlu et al. [70] found a significant increase in gut microbial diversity and richness in female with PCOS following kefir consumption, possibly due to the exclusion of participants using antibiotics. This collective data indicates the impact of kefir consumption on the gut microbiome is highly dependent on host health and lifestyle factors.

Despite variation across studies, several common findings emerged. Three studies reported a link between increased LAB levels and improvements in health outcomes. Yılmaz et al. [74] observed an increase in *Lactobacillus* levels in individuals with IBD, correlating with improvements in gastrointestinal symptoms, particularly in the ‘abdominal pain’ and ‘feeling good’ scores. Similarly, Gupta et al. [71]. reported an increase in *Lactobacillus* as being linked to improved GMWI in critically ill adults. However, this increase was for a brief period of only 72 h during the study. Dazıroğlu et al. [70]. found a significant rise in *Bacilli* abundance in female with PCOS, as well as a statistically significant improvement in physical function and mental health scores compared to pre-treatment. Two studies provided some preliminary evidence that increased LAB could improve health, although clear links were not established. Walsh et al. [72] identified an increase in *L. raffinolactis*, though this was only in four of the ten participants in the kefir group. These four participants demonstrated increases in acetic acid, N, N-dimethylglycine and succinic acid in urine, metabolites which may support metabolism, muscle health and skin health [79,80,81]. However, specific changes in the health outcomes of these four participants were not reported. Bellikci-Koyu et al. [73] reported a minor increase in *Lactobacillales* in individuals with metabolic syndrome and positive changes in fasting insulin, TNF-a, IFN-y and blood pressure in the kefir group. However, changes in overall health outcomes were also not reported. This indicates that consumption of kefir, by initiating changes in the abundance of LAB in the gut microbiome, may lead to positive changes in gut health and systemic health. However, this was not reflected in the study by Öneş et al. [69]. However, it was found that minor improvements in athletic performance occurred following kefir consumption, though this was not statistically significant. These studies report associations rather than causal relationships, and there is little homogeneity between studies regarding improved health measures.

Among the reviewed studies, three studies reported using kefir from different manufacturers [69,71,72], two reported formulating their own kefir from different manufacturers [70,73], and one did not report the manufacturer of the kefir used [74]. Standardised sources would enable more reliable cross-study comparisons. The inclusion of placebo controls in study designs helps to ensure the validity of results, yet only one study compared the kefir intervention group with a control group receiving a placebo treatment [73]; two studies included a control group that received no treatment [69,74], and the remaining three studies did not include a control group [70,71,72]. Larger-scale trials are also needed, given the limited sample sizes across all included studies.

### 4.2. Changes in the Oral Microbiome Induced by Kefir Consumption

Across all studies, kefir consumption in humans appeared to reduce the abundance of specific oral microbiota. All four studies reported reduced colony-forming units (CFU) of salivary *Streptococcus mutans*, a key contributor to dental caries, following a period of kefir consumption [75,76,77,78]. Cogulu et al. [78] also reported reduced CFU of salivary *Lactobacillus*, which includes species that both contribute to and prevent dental caries. This finding was not reflected in the remaining three studies. However, the general response to kefir consumption appears consistent across age groups. Two studies focused on healthy adults, demonstrating reductions in cariogenic bacteria *S. mutans* [77,78]. The remaining two studies involved children undergoing orthodontic treatment or dental restorations and demonstrated the same reduction [75,76]. This suggests that regardless of age or dental health status, kefir consumption may reduce the prevalence of cariogenic bacteria. However, due to the limited outcomes and the lack of long-term follow-ups across all four studies, there is no evidence to confirm that kefir consumption directly reduces the risk of dental caries or other dental diseases via modulation of the oral microbiome. Further studies should include assessment of clinical outcomes via long-term follow-ups to further elucidate the link between kefir consumption and the support of overall oral health.

While reductions in salivary *S. mutans* and *Lactobacillus* were reported across the four studies, these were the only observed changes in the oral microbiome. This represents a critical limitation of the literature, as all four studies relied exclusively on culture-based methods to identify bacteria present in participant salivary samples. None of the four reported studies used DNA sequencing methods to identify bacteria; instead, culturing methods and Caries risk tests (CRT) were used [75,76,77,78]. CRT involves collecting patient saliva and using an in-built agar media to visually determine the abundance of *S. mutans* and *Lactobacilli* present in saliva. As these methods are semi-quantitative and only test for the presence of pre-determined bacteria, they cannot provide a comprehensive view of microbial diversity, nor can they identify non-predetermined and novel species; this can only be achieved via DNA sequencing methods. Thus, the sole reliance on culture-based identification methods across all four studies majorly precludes comprehensive assessment of kefir consumption’s broader effects on oral microbial ecology.

Other limitations of the four studies include the varied kefir product manufacturers. One study used kefir grains from an unidentified manufacturer to produce a kefir product given to participants [77], two studies used different sources [76,78], and one did not report on the manufacturer of the kefir used [75]. Another limitation is the relatively small cohorts; only two trials included over 50 participants [76,77], with one of these not reporting the participants’ allocations to respective groups [77]. Larger-scale trials are needed to ensure reliable results. Furthermore, there was no evidence across the four studies of strict control of confounding factors, such as participant oral hygiene and dietary habits. While Alp et al. [76] state that participants received oral hygiene education and were advised to brush twice daily, details regarding the nature and content of the education were not included, and participant compliance with recommended oral hygiene practices was not recorded. Similarly, Reddy et al. [75] directed participants to maintain their usual hygiene practices, yet there is no evidence to suggest that authors monitored compliance or adjusted for its potential effects. Finally, the two remaining studies did not specify any instructions regarding participants’ oral hygiene practices [77,78]. These studies also employed intervention periods of three weeks or less [77,78], a short intervention period relative to other studies on the gut and oral microbiome. This short period may not be sufficient to allow changes in the oral microbiome to occur following kefir consumption. The short intervention period therefore stands as a major limitation for these two studies.

## 5. Conclusions

The current literature indicates that kefir consumption may influence both the gut and oral microbiomes in humans, but the specific nature and magnitude of these effects differ. In the gut, kefir appears to influence the relative abundance of *Lactobacillus*, with some studies also showing improvements in wellness indices and clinical parameters. In the oral cavity, kefir consistently reduces levels of *Streptococcus mutans*, a primary contributor to dental caries; a result observed in both adult and child populations. However, the variations in study designs and outcome measures makes it difficult to draw definitive conclusions about the specific mechanisms and long-term clinical implications of kefir consumption.

## 6. Future Directions

Future research should aim to overcome the multiple limitations present in the current body of studies. Recurring issues such as small sample sizes, brief intervention periods, and the absence of appropriate controls undermine the translation of the data. Additionally, variations in kefir grain sources or kefir manufacturers across the studies may introduce confounding factors that influence study results. In oral microbiome studies, the reliance on culture-based methods and Caries risk tests, rather than next-generation sequencing, severely restricted the scope of research to changes in the count of pre-determined bacteria. As a result, changes in alpha or beta diversity and relative abundance were not assessed. Future research should address these gaps through larger, well-controlled trials with age-, sex-, and ethnicity-matched participants using standardized kefir products, well-described kefir bases (dairy vs. plant-based) and comprehensive microbiome profiling. Additionally, longitudinal studies are needed to evaluate kefir’s long-term impact on clinical outcomes, including dental caries incidence and gut-related disorder prevention or management. Addressing these limitations would provide a more comprehensive understanding of kefir’s health benefits and therapeutic potential.

## Figures and Tables

**Table 1:** Comparison of six studies investigating the effect of kefir consumption on gut microbiota in humans. Abbreviations: ns = not statistically significant as reported in identified study; GMWI = Gut Microbiome Wellness Index; T1 = timepoint 1; T2 = timepoint; PCOS = polycystic ovarian syndrome; LOS = length of stay; CD = Crohn’s disease; UC = ulcerative colitis; NR = not reported.

Reference, Country	Participants	Treatments	Dosage	Treatment Duration	Kefir Product and Substrate Used	Sampling Methods	Identification Methods	Key Findings	Limitations
Öneş et al., 2025 [69] Turkey	Professional female soccer players *n* (intervention) = 12 *n* (control) = 9 Age(intervention) = 24.42 ± 2.52 years Age (control) = 22.14 ± 3.61 years Gender (intervention) = 100% female Gender (control) = 100% female	Intervention received kefir; control received nothing.	200 mL once daily	4 weeks	Altınkılıç Lactose-free Kefir, cow’s milk	External faecal sample processed using DiaRex stool senomic DNA extraction kit	V3–V4 region of 16S rRNA	↑ Microbial richness and diversity in intervention group only (ns) ↑ Finishing speed and VO_2_max (ns) Minor changes in relative abundance in intervention group only (ns)	Short duration; small sample size; no placebo control; no wash-out period; no analysis of dose-dependent effects; specific population
Dazıroğlu et al., 2024 [70] Turkey	Females with PCOS *n* (intervention) = 17 Age (intervention) = 24.7 ± 5.44 years Gender (intervention) = 100% female	Participants received kefir	250 mL once daily	8 weeks	Authors formulated their own kefir through traditional practices using grains from Danem, substrate unclear	External faecal sample processed using DiaRex stool genomic DNA extraction kit	V3-V4 region of 16S rRNA	↓ IL-6 ↑ *Bacilli* ↑ *Lactococcus* ↑ *Holdemania*	Small sample size; no placebo control; no wash-out period; no analysis of dose dependent effects
Gupta et al., 2024 [71] USA	Critically ill males and females *n* (intervention) = 54 *n* (microbiota analysis) = 13 Age (intervention) = 64.6 ± 15.3 years Gender (intervention) = 39% female	Patients received kefir	60 mL, followed by 120 mL after 12 h, then 240 mL daily	Until discharge of patient	Lifeway Foods Kefir, whole cow’s milk	External faecal sample processed using Qiagen DNeasy 96 PowerSoil pro QIAcube HT kit	Shotgun metagenomic sequencing	↑ *Lactobacillus plantarum*, *L. reuteri*, and *L. rhamnosus* from T1 to T2 ↑ GMWI scores in critically ill adults. ↓ Shannon diversity and species richness Administration was safe and feasible	Small sample size; no placebo control; no wash-out period; no analysis of dose-dependent effects; duration of intervention limited by LOS and death; high rate of antibiotic use
Walsh et al., 2023 [72] Ireland	Healthy individuals *n* (inulin) = 10 *n* (probiotic) = 10 *n* (kefir) = 9 Age (inulin) = NR Age (probiotic) = NR Age (kefir) = NR Gender (inulin) = NR Gender (probiotic) = NR Gender (kefir) = NR	Inulin group received inulin; kefir group received kefir; probiotic group received probiotic	247 mL once daily	4 weeks	Nourish Kefir, substrate unclear	External faecal sample processed using Qiagen QIAamp DNA stool mini kit	Shotgun metagenomic sequencing	↑ *Lactococcus raffinolactis* in kefir group only (ns)	Short duration; small sample size; no placebo control; no wash-out period; no analysis of dose-dependent effects
Bellikci-Koyu et al., 2019 [73] Turkey	Males and females with metabolic syndrome *n* (intervention) = 12 *n* (control) = 10 Age (intervention) = 52 (range 47.5–60.5) Age (control) = 53 (range 45–60) Gender (intervention) = 83% female Gender (control) = 60% female	Intervention received kefir; control received unfermented milk	180 mL once daily	12 weeks	Authors formulated their own kefir using grains from Danisco, full-fat whole cow’s milk	External faecal sample processed using Qiagen QIAamp DNA stool mini kit	V3-V4 region of 16S rRNA	↑ *Actinobacteria*; ↑ *Lactobacillales* (ns) Positive changes in fasting insulin, TNF-a, IFN-y and blood pressure in kefir group (ns between groups)	Small sample size; no wash-out period; no analysis of dose-dependent effects; dietary confounding; no mechanistic explanation for changes in biological parameters studied
Yılmaz et al., 2019 [74] Turkey	Individuals with IBD *n* (intervention, CD) = 10 *n* (intervention, UC) = 15 *n* (control, CD) = 10 *n* (control, UC) = 10 Age (intervention, CD) = 33 (range 24–65) Age (intervention, UC) = 33 (range 19–68) Age (control, CD) = 42 (range 21–66) Age (control, UC) = 43.5 (range 29–76) Gender (intervention, CD) = 60% female Gender (intervention, UC) = 40% female Gender (control, CD) = 40% female Gender (control, UC) = 60% female	Intervention groups received kefir; control received nothing.	400 mL twice daily	4 weeks	NR, substrate unclear	External faecal sample processed using Qiagen QIAamp DNA stool mini kit	Quantitative polymerase chain reaction and Sanger sequencing	↑ *Lactobacillus* (CD/UC patients in kefir group only)	Short duration; small sample size; no placebo control; no wash-out period; no analysis of dose-dependent effects; analysis limited to *Lactobacillus*; statistically significant differences in levels of faecal *Lactobacillus* between UC control and UC treatment groups at baseline

↑: increased; ↓: decreased.

**Table 2:** Comparison of six studies investigating the effect of kefir consumption on oral microbiota in humans. Abbreviations: NR = not reported; CFU = colony forming units.

Reference, Country	Participants	Treatments	Dosage	Treatment Duration	Kefir Product and Substrate Used	Sampling Methods	Identification Methods	Key Findings	Limitations
Reddy et al., 2021 [75] India	Children with carious lesions post-restoration *n* = 80 *n* (kefir) = NR *n* (probiotic curd) = NR *n* (probiotic drink) = NR *n* (control) = NR Age (kefir) = NR Age (probiotic curd) = NR Age (probiotic drink) = NR Age (control) = NR Gender (kefir) = NR Gender (probiotic curd) = NR Gender (probiotic drink) = NR Gender (control) = NR	Kefir group received kefir; probiotic curd group received probiotic curd; probiotic drink group received probiotic drinks; control group received nothing.	100 mL once daily	4 weeks	NR, substrate unclear	Unstimulated saliva sample	Culture-based identification	↓ Salivary *Streptococcus mutans* in kefir compared to the control group at 4 weeks.	Group allocation NR; no wash-out period; limited outcome measures; no analysis of dose-dependent effects; dietary and behavioural confounding; control group had higher CFU *Streptococcus mutans* at baseline.
Alp et al., 2018 [76] Turkey	Children undergoing orthodontic treatment *n* (kefir) = 15 *n* (probiotic toothpaste) = 15 *n* (control) = 15 Age (kefir) = 14.3 ± 1.7 years Age (probiotic toothpaste) = 14.9 ± 2.0 years Age (control) = 14.1 ± 2.1 years Gender (kefir) = 47% female Gender (probiotic toothpaste) = 67% female Gender (control) = 67% female	Kefir group received kefir; probiotic toothpaste group brushed with probiotic toothpaste; control group brushed with regular toothpaste	100 mL twice daily	6 weeks	Atatürk Orman Çiftliği (Atatürk Forest Farm) Kefir, substrate unclear	Stimulated saliva samples	Caries risk test	↓ Salivary *Streptococcus mutans* in kefir and probiotic toothpaste groups compared to control at 3 weeks and 6 weeks.	Short duration; small sample size; no wash-out period; limited outcome measures; no analysis of dose-dependent effects; dietary and behavioural confounding.
Ghasempour et al., 2014 [77] Iran	Healthy individuals *n* = 22 *n* (Group A) = 11 *n* (Group B) = 11 Age (Group A) = NR Age (Group B) = NR Gender (Group A) = NR Gender (Group B) = NR	Crossover design. Participants in group A received kefir for two weeks, then 0.05% sodium fluoride for two weeks. Participants in group B received the same treatments in a reverse-parallel manner.	100 mL once daily	2 weeks	Authors formulated their own kefir, although did not specify manufacturer of grains used, cow’s milk.	Unstimulated saliva sample	Culture-based identification	↓ Salivary *Streptococcus mutans* in intervention and control compared with baseline after 2 weeks. Equal inhibitory effect was found between study groups.	Short duration; small sample size; no analysis of baseline characteristics; limited outcome measures; no analysis of dose-dependent effects; dietary confounding.
Cogulu et al., 2010 [78] Turkey	Healthy individuals *n* (intervention, single dose) = 35 *n* (intervention, double dose) = 35 *n* (control) = 34 Age (intervention single dose) = NR Age (intervention double dose) = NR Age (control) = NR Gender (intervention single dose) = NR Gender (intervention double dose) = NR Gender (control) = NR	Single dose group received kefir; double dose received twice the amount of kefir twice daily for 3 weeks; control group received 100 mL milk once daily for 3 weeks.	100 mL; once daily for single-dose, twice daily for double-dose	3 weeks	Sakipaga Kefir, substrate unclear	Stimulated saliva samples	Caries risk test	↓ Salivary *Streptococcus mutans* in double-dose compared to single-dose and control after 3 weeks. ↓ Salivary *Lactobacillus* in double-dose compared to single-dose and control after 3 weeks.	Short duration; no wash-out period; limited outcome measures; dietary and behavioural confounding.

↓: decreased.

## Data Availability

Data is contained within the article. The original contributions presented in this study are included in the article. Further inquiries can be directed to the corresponding author.
